# A Point Mutation in a lincRNA Upstream of *GDNF* Is Associated to a Canine Insensitivity to Pain: A Spontaneous Model for Human Sensory Neuropathies

**DOI:** 10.1371/journal.pgen.1006482

**Published:** 2016-12-29

**Authors:** Jocelyn Plassais, Laetitia Lagoutte, Solenne Correard, Manon Paradis, Eric Guaguère, Benoit Hédan, Alix Pommier, Nadine Botherel, Marie-Christine Cadiergues, Philippe Pilorge, David Silversides, Maud Bizot, Mark Samuels, Carme Arnan, Rory Johnson, Christophe Hitte, Gilles Salbert, Agnès Méreau, Pascale Quignon, Thomas Derrien, Catherine André

**Affiliations:** 1 CNRS, UMR 6290, Institut de Génétique et Développement de Rennes, Rennes, France; 2 Université Rennes 1, UEB, Biosit, Faculté de Médecine, Rennes, France; 3 Department of Clinical Sciences, Faculté de Médecine Vétérinaire, University of Montreal, Montreal, Québec, Canada; 4 Clinique Vétérinaire Saint Bernard, Lomme, France; 5 UDEAR, Université de Toulouse, Ecole Nationale Vétérinaire de Toulouse, INSERM, Toulouse, France; 6 Clinique Vétérinaire de Saint-Cyr, Rennes, France; 7 Department of Biochemistry and Molecular Medicine, CHU Sainte-Justine, University of Montreal, Montreal, Québec, Canada; 8 Centre for Genomic Regulation (CRG), Barcelona, Spain; 9 Universitat Pompeu Fabra (UPF), Barcelona, Spain; 10 Institut Hospital del Mar d’Investigations Mèdiques (IMIM), Barcelona, Spain; Cornell University College of Veterinary Medicine, UNITED STATES

## Abstract

Human Hereditary Sensory Autonomic Neuropathies (HSANs) are characterized by insensitivity to pain, sometimes combined with self-mutilation. Strikingly, several sporting dog breeds are particularly affected by such neuropathies. Clinical signs appear in young puppies and consist of acral analgesia, with or without sudden intense licking, biting and severe self-mutilation of the feet, whereas proprioception, motor abilities and spinal reflexes remain intact. Through a Genome Wide Association Study (GWAS) with 24 affected and 30 unaffected sporting dogs using the Canine HD 170K SNP array (Illumina), we identified a 1.8 Mb homozygous locus on canine chromosome 4 (adj. p-val = 2.5x10^-6^). Targeted high-throughput sequencing of this locus in 4 affected and 4 unaffected dogs identified 478 variants. Only one variant perfectly segregated with the expected recessive inheritance in 300 sporting dogs of known clinical status, while it was never present in 900 unaffected dogs from 130 other breeds. This variant, located 90 kb upstream of the *GDNF* gene, a highly relevant neurotrophic factor candidate gene, lies in a long intergenic non-coding RNAs (lincRNA), *GDNF-AS*. Using human comparative genomic analysis, we observed that the canine variant maps onto an enhancer element. Quantitative RT-PCR of dorsal root ganglia RNAs of affected dogs showed a significant decrease of both *GDNF* mRNA and *GDNF-AS* expression levels (respectively 60% and 80%), as compared to unaffected dogs. We thus performed gel shift assays (EMSA) that reveal that the canine variant significantly alters the binding of regulatory elements. Altogether, these results allowed the identification in dogs of *GDNF* as a relevant candidate for human HSAN and insensitivity to pain, but also shed light on the regulation of *GDNF* transcription. Finally, such results allow proposing these sporting dog breeds as natural models for clinical trials with a double benefit for human and veterinary medicine.

## Author Summary

In this study, we present a canine neuropathy characterized by insensitivity to pain in the feet, sometimes combined with self-mutilation described in four sporting breeds. This particular phenotype has the clinical hallmarks of human Hereditary Sensory Autonomic Neuropathies (HSAN). As we hypothesized that a monogenic recessive disorder was shared between these breeds, we performed a Genome Wide Association Study (GWAS) to search for the genetic causes and found one homozygous chromosomal region in affected dogs. High-throughput sequencing of this region allowed the identification of a point mutation upstream to the *GDNF* gene and located in the last exon of a long non-coding RNA, *GDNF-AS*. We confirmed the perfect association of this variant with the disease using more than 900 unaffected dogs that do not present with this mutation. Functional analyses (qRT-PCR, EMSA) confirmed that the mutation alters the binding of regulatory complex, leading to a significant decrease of both *GDNF* and *GDNF-AS* mRNA expression levels. This work in canine spontaneous forms of human neuropathies allowed the identification of a novel gene *GDNF* and its regulation mechanism, not yet described in human HSAN, opening the field of clinical trials to benefit both canine and human medicine.

## Introduction

Inherited peripheral neuropathies are neurodegenerative diseases of the peripheral nervous system (PNS), including the Charcot-Marie-Tooth diseases (CMT) and Hereditary Sensory and Autonomic Neuropathies (HSANs) [1]. They are categorized based on the degree of involvement of motor, sensory and/or autonomic nerve fibers. HSANs are rare peripheral neuropathies constituting a clinically and genetically heterogeneous group which phenotypes range from pure sensory involvement to variable levels of motor and autonomic disturbances [2]. The HSAN types can be subdivided into two groups based on their mode of inheritance, HSAN I being autosomal dominant disorders and HSAN II to V being recessive. The main symptoms correspond to a progressive degeneration of sensory and autonomic neurons causing insensitivity to pain and temperature in feet and hands, leading to ulcerative mutilations [3–5]. These lesions could result in severe tissue infections and/or osteomyelitis and may lead to amputation of the affected limbs. To date, the pathophysiologic mechanisms known to be implicated in HSAN include sphingolipid metabolism with mutations in *SPTCL1* or *SPTCL2* (serine palmitoyl-transferase genes), vesicular transport (*RAB7*, *IKBKAP*) or neurotrophic factors and their tyrosine kinase receptors (*NTRK1*, *NGF-B*) [6–8]. Although 12 genes have been associated with HSANs until now, they only explain one third of the patients affected with HSANs, highlighting the fact that the genetic causes of HSANs remain only partially understood in humans [2] and that natural models of these diseases are needed.

**Fig 1:**
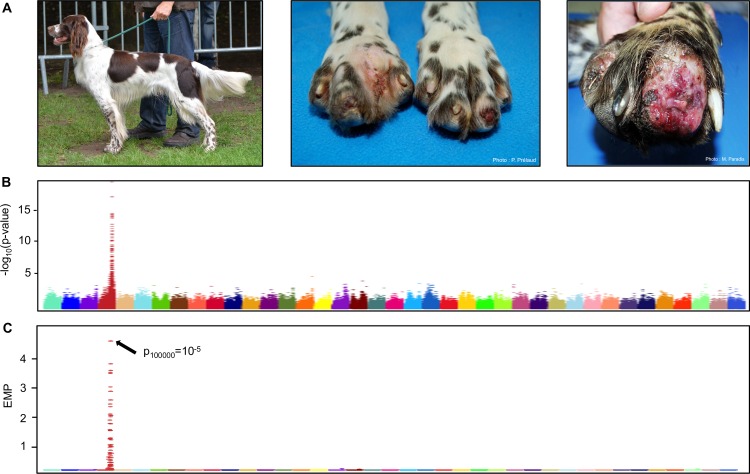
Association study for the acral mutilation syndrome in sporting dogs. (A) Clinical phenotype in the French spaniel breed (left picture). Dogs present insensitivity to pain in feet and can sometimes show severe self-mutilations with the absence of a toe. (B) Manhattan plot of -log10 transformed p-values by canine chromosome highlighting a strong signal on chromosome 4 (Wald test; p ≤ 10^−16^). C) Manhattan plot of -log10 empirical p-values (EMP) obtained by permutations test confirming the signal on chromosome 4 (Permutation = 100,000).

Interestingly, while neurological diseases such as HSANs are rare in humans, some dogs breeds spontaneously develop such diseases with high frequencies [9]. Indeed, recent breeding practices have created genetically isolated populations with large homozygous haplotypes and linkage disequilibrium (LD) blocks [10–12]. With this particular genetic structure of population, the dog model allowed the identification of new genes involved in rare diseases [13,14], and thus constitutes an excellent model to study sensory neuropathies. Indeed, a large number of inherited motor and sensory neuropathies were reported in the dog species [9,15]. Since 1960’s, symptoms similar to human HSANs were described in four sporting breeds, with the first cases reported in German short-haired Pointers (GP) in 1964 [16,17], then in English Pointers (EP), English Springer Spaniel (ESS) and more recently in French Spaniel (FS) [16,18–21]. For these four breeds, the hallmarks of the disease are loss of pain sensation and feet ulceration. Symptoms naturally occur in puppies approximately four months old when they begin to lick and bite their paws. Affected dogs present an acral insensitivity to pain with, in the majority of cases, severe self-mutilations of the feet including claw loss, painless fractures, and digit amputation (Fig 1A). No sign of autonomic disturbances are detected and motor abilities and proprioception are normal. The pathologic process thus seems to only affect sensory neurons, primarily their development, followed by progressive postnatal degeneration of unmyelinated fibers [18–20]. In addition, pedigree analyses in these breeds revealed a recessive autosomal mode of inheritance such as in human type II-V HSANs [2,21].

We thus investigated these affected breeds as a potential opportunity to identify new genes for unexplained cases of human HSANs. We first started the genetic study with the French Spaniel breed and identified one locus strongly associated with the disease, on canine chromosome 4, and refined the locus by intersecting the haplotypes of the other affected breeds. Using this strategy, we highlighted a common homozygous haplotype of 1.8 Mb (chr4:70,6–72,4Mb) shared by all affected dogs in the four sporting breeds. Next, we performed targeted DNA sequencing of the locus in four affected and four unaffected dogs. We found 478 variants which were individually removed by Allele-Specific PCR except one located upstream of the *GDNF* gene (*Glial cell-Derived Neurotrophic Factor*), a neurotrophic factor involved in neuronal development and adult neuronal survival [22–25]. More precisely, we showed that this variant is localized in the last exon of a canine long-non-coding RNA (*GDNF-AS*) that is transcribed in the opposite direction of *GDNF*. Comparative genomic analysis with the human orthologous genomic region led us to hypothesize that the mutation disrupts a regulatory region, such as an enhancer, controlling *GDNF* expression. We then performed quantitative PCR analyses and showed lower expression levels of both *GDNF* and *GDNF-AS* RNAs in affected dogs compared to unaffected dogs. By gel shift assays, we further showed that the mutation modified the affinity of a regulatory complex. Such findings highlight the *GDNF*/*GDNF-AS* partnership as a subtle regulatory mechanism and relevant candidates to search for human HSAN mutations and potentially to develop targeted therapies.

## Results

### Genome-wide association study and *GDNF* sequencing

We first focused the genetic analysis on the French Spaniel breed. The collection of 173 blood samples with pedigree information allowed us to design a large pedigree and to confirm the monogenic recessive inheritance of the disease in this breed as previously described [21]. Affected dogs presented with severe self-mutilations with insensitivity to pain only in the feet (Fig 1A). Neurological examinations (proprioception, motor abilities, spinal reflex exams) confirmed that pain perception became progressively normal above the knees and was not altered in the rest of the body. One affected dog had never shown self-mutilation marks leading us to consider insensitivity to pain as the first clinical sign for this disease. As described in Paradis *et al*, no signs of autonomic disturbances were detected by neurological exams [21] and motor abilities and proprioception were normal in all dogs.

**Fig 2:**
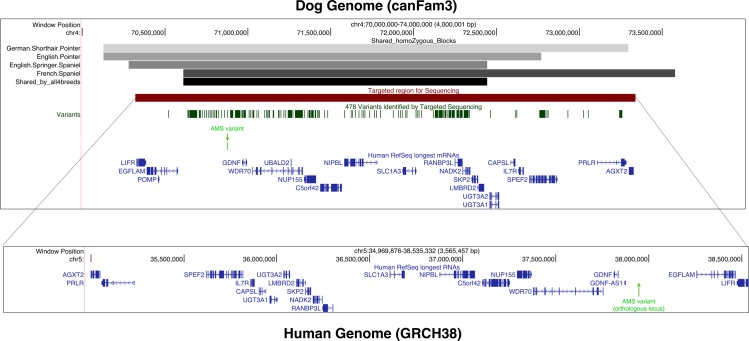
UCSC screenshot of the dog chromosome 4 locus (CanFam3) found by GWAS and its orthologous genomic region in human (GRCh38). The upper panel represents the canine locus with the first track showing homozygous haplotype blocks found in four sporting breeds (shaded greys) and the bottom horizontal bar corresponding to the common 1.8 Mb locus shared by the four breeds (black). Next tracks show the 3 Mb region targeted for high throughput sequencing (in red) and the resulting 478 variants identified by GATK program (dark green). The AMS variant is represented as a vertical bar (light green) and highlighted by a green arrow. Finally, the non-dog RefSeq genes (longest isoform) are represented in blue. The bottom panel corresponds to the human orthologous region (inverted orientation) of the canine targeted interval as given by the LiftOver tools available on the UCSC genome browser [26,27]. Human RefSeq genes are annotated in blue and the predicted orthologous genomic position of the AMS variant corresponds to the green arrow.

We first performed a genome-wide association study (GWAS) using a linear mixed-model method accounting for population stratification and relatedness, with 49 French spaniels (21 affected showing insensitivity to pain in their feet and 28 unaffected dogs) (Fig 1B). Following quality control of the genotyping data, 123,579 SNPs were retained for genetic mapping. This analysis identified a 3 Mb locus on canine chromosome 4 strongly associated with the disease, with significant p-values (Wald test; p-value = 10^−16^; Table 1) and confirmed the association using a permutation test (p_100,000_ = 10^−5^) (Fig 1C). Since all affected French spaniels present a homozygous haplotype in this 3 Mb locus, we took advantage of the particular genetic structure of the dog population. Based on the hypothesis that sporting breeds share the same disease and founder mutation, we genotyped additional affected dogs in three sporting dog breeds: German Short-haired Pointer (GP), English Pointer (EP) and English Springer Spaniel (ESS). These breeds revealed a common homozygous haplotype block with French Spaniel (FS) confirming the founder effect between these four breeds. Recombination breakpoints in the FS and ESS reduced the critical interval to 1.8 Mb (chr4:70,6–72,4Mb) (Fig 2). This interval contains a dozen genes annotated in the dog and human orthologous genomic region including the *GDNF* gene (*Glial cell-Derived Neurotrophic Factor*), which appeared as a new and good candidate for HSAN (Table 1). Indeed, *GDNF* codes a neurotrophic factor involved in neuronal development and adult neuronal survival [22–25] and was never associated with HSAN. However, Sanger sequencing did not reveal any mutation neither in the three exons of the transcripts, nor in the promoter sequence of *GDNF*.

**Table 1:** Best-SNPs obtained by GWAS using 21 affected *vs* 28 unaffected French spaniels.

Chromosome	SNP position	Reference Allele	Rare Allele	Minor Allele Frequency	P-Wald	Closest gene
Chr4	70687820	C	T	0,337	1,10E-16	*GDNF*
Chr4	72114166	A	G	0,337	1,10E-16	*SLC1A3*
Chr4	73168208	T	C	0,337	1,10E-16	*PRLR*
Chr4	72094031	T	C	0,348	2,09E-16	*SLC1A3*
Chr4	72313249	G	A	0,348	2,09E-16	*NADK2*
Chr4	72330521	G	A	0,348	2,09E-16	*NADK2*
Chr4	72336552	A	G	0,348	2,09E-16	*NADK2*
Chr4	72634107	C	T	0,348	2,09E-16	*IL7R*
Chr4	72635293	C	T	0,348	2,09E-16	*IL7R*
Chr4	72722983	G	A	0,348	2,09E-16	*SPEF2*

### Targeted re-sequencing of the associated locus and validation of the mutation

To identify the disease-associated variants (InDel and SNP), we chose to enlarge the homozygous candidate region to 3 Mb (70.3 Mb to 73.3 Mb on CanFam3 assembly) and performed a targeted sequencing (Fig 2). Four affected and four unaffected sporting dogs were thus sequenced with high coverage and depth (coverage 25X: 99%; 10 X: 100%; mean depth: 374X; S1 Table). Within this 3 Mb interval, 478 variants were identified with the expected distribution between affected and unaffected individuals (Tables 2 and 3). After QC and filtering for known SNPs from the dbSNP database (dbSNP build 139), 156 variants still remained (Table 4 and S2 Table). To exclude non-disease variants due to polymorphisms, we then chose to genotype dogs from a large number of unaffected breeds making the hypothesis that the rare allele present in affected dogs should not be found in unaffected breeds (S3 Table). Using AS-PCR experiment and Sanger sequencing, we found only one SNP perfectly segregating with the disease and located in an intergenic region ~90 kb upstream of the *GDNF* gene (chr4.g.70,875,561C>T). To validate this variant, we sequenced over 900 unaffected dogs from 130 different breeds and observed that the mutated allele was never detected in these dogs, thus excluding a polymorphism. We also checked the allelic distribution of this variant in a panel of 250 sporting dogs of known clinical status and we observed a perfect correlation between the clinical status of dogs and their genetic status, confirming the strong association of the variant with the disease in the analyzed sporting breeds. Interestingly, heterozygous dogs did not present any clinical signs and only one dog was homozygous mutated with only insensitivity to pain (no acral mutilations). Even if it was lying in a not yet described genomic region, we considered this variant as potentially causal and further named it “AMS variant” for Acral Mutilation Syndrome variant.

**Table 2:** Summary of the SNPs found in the targeted sequencing. Only variants that are homozygous for a rare allele in all affected dogs and not homozygous in controls were considered to perfectly segregate. Nb SNPs: Number of SNPs; Hom: Nb Homozygous variants; HTZ: Nb Heterozygous variants.

Sample	Nb SNPs	Hom	HTZ	3.UTR	5.UTR	Non sense	Missense	Synonymous	Intronic	OutGene	miRNA
French Spaniel-Control 1	2765	1152	1613	15	88	0	19	21	1319	1326	0
French Spaniel-Case 1	2447	2409	38	16	86	0	14	27	1311	1014	0
French Spaniel-Case 2	2533	2270	263	16	84	0	15	28	1371	1039	0
English Springer Spaniel-Case	2506	2470	36	16	75	0	14	21	1299	1102	0
English Springer Spaniel-Control	2857	2176	681	17	149	0	20	26	1446	1219	0
French Spaniel-Control 2	3098	1092	2006	16	102	0	19	30	1606	1348	0
German Shorthaired Pointer-Control	2472	2434	38	16	86	0	14	27	1313	1036	0
German Shorthaired Pointer-Case	3451	1319	2132	24	125	0	20	31	1786	1488	0

**Table 3:** Summary of the indels found in the targeted sequencing. Only variants that are homozygous for a rare allele in all affected dogs and not homozygous in controls were considered to perfectly segregate. Hom: Nb Homozygous variants; HTZ: Nb Heterozygous variants.

Sample	Insertion	Deletion	Hom	HTZ	3.UTR	5.UTR	Coding	Intronic	OutGene
French Spaniel-Control 1	427	393	309	534	6	31	3	413	385
French Spaniel-Case 1	385	392	685	117	4	34	3	407	355
French Spaniel-Case 2	391	405	669	158	4	36	3	424	363
English Springer Spaniel-Case	386	388	673	129	4	27	2	396	368
English Springer Spaniel-Control	431	399	617	243	8	43	3	448	363
French Spaniel-Control 2	481	467	290	689	5	35	3	500	427
German Shorthaired Pointer-Control	392	395	681	135	3	31	3	416	360
German Shorthaired Pointer-Case	499	538	336	735	6	46	4	529	467

**Table 4:** Summary of the variants obtained in the targeted sequencing before and after filtering.

	Before filtering	After filtering (QC and known in dbSNPs)
Variants that segregate perfectly across 8 samples	478	156
Number of candidates in coding elements (missense, indels)	3	1

### Genomic analyses of the chromosome 4 locus

To unravel the genomic context surrounding the *GDNF* locus, we mapped the canine 3 Mb locus targeted for sequencing (chr4:70,3–73,3 Mb) onto the human genome (GRCh38) using the LiftOver tools available on the UCSC genome browser [26,27] and the AutoGRAPH website [28] (Fig 2). The locus is well conserved between dog and human with 63.3% sequence identity with human region (chr5:34,9 Mb-38,5 Mb) and 53.1% reciprocally. At the syntonic level, most of the canine orthologous protein-coding genes harbor the same order but in the opposite orientation of transcription as compared to humans. Remarkably in human, a long non-coding RNA gene (*GDNF-AS*) is annotated upstream of *GDNF* [29], transcribed in a divergent orientation, and close to the orthologous position of the canine AMS variant (Fig 2).

**Fig 3:**
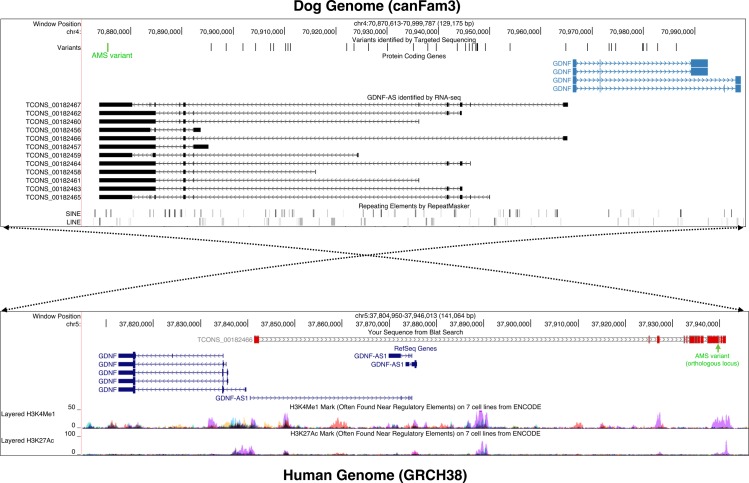
UCSC screenshots of the candidate *GDNF/GDNF-AS* regions in dog (CanFam3 assembly) and in human (GRCh38 assembly). The upper panel represents the canine *GDNF/GDNF-AS* locus (chr4:70,8–71 Mb) with a subset of the variants identified by targeted sequencing in black and the AMS variant highlighted in green, the *GDNF* mRNA isoforms annotated by the BROAD institute [32] (in light blue), the mapping of the 12 canine *GDNF-AS* isoforms identified in our RNA-seq analysis (in black), and the RepeatMasker track of SINEs and LINEs in the locus (shaded gray). The bottom panel represents the human orthologous locus (chr5:37,8–37,9 Mb) with tracks corresponding (from the top to bottom) to the alignment of the canine *GDNF-AS* isoform (TCONS_00182466) using BLAT [26] (in grey and red) with the orthologous position of the AMS variant (in green), the RefSeq genes (in blue) and the H3K4me1 and H3K27ac chromatin signals annotated by the ENCODE project [36] with NHEK cell line in purple. The dotted arrows illustrate the inversion of the syntonic block between dog and human species.

To refine the annotation of the canine locus, we used 33 RNA sequencing data produced from 25 different tissues of seven breeds (S4 Table). The sequencing data were provided by the Broad Institute and as part of a collaborative work of the LUPA consortium [30–32]. This extended annotation confirmed the presence of a multi-exonic RNA transcribed in the vicinity and in a divergent orientation of *GDNF* as in human (Fig 3). Next, to assess the protein-coding capabilities of each of the 12 isoforms of the new gene, we used three complementary programs (CPC [33], CPAT [34], PLEK [35]) and confirmed that all of the new transcripts are classified as probable non-coding RNAs (See Methods and S1 File). Remarkably, the AMS variant is located in the last exon of the canine lincRNA, which is shared by all of the 12 isoforms (Fig 3). The lincRNA, that we named canine *GDNF-AS*, is weakly expressed in the 33 RNA-seq (S5 Table) and is mostly detected in the olfactory bulb (RPKM = 0.849) and spleen (RPKM = 0.732). We also observed that the highest level of *GDNF* mRNA expression level is in spleen (RPKM = 4.6).

It has been shown that lincRNAs transcribed from a bi-directional promoter could regulate the level of expression of the corresponding neighbor mRNAs [37–39]. We therefore computed the correlation of expression using the 33 expression data values (RPKM) between the canine *GDNF-AS* and the four closest protein-coding genes *i*.*e*. *GDNF*, *WDR70*, *NUP155* and *C5orf42* (S1 Fig). We found that *GDNF-AS* expression is highly correlated with *GDNF* (Pearson correlation = 0.67, p-value = 1.8 10^−5^) but not with the other protein-coding genes (Pearson correlations = -0.10, -0.03 and 0.08 for *WDR70*, *NUP155* and *C5orf42*, respectively). These results, together with the close proximity of *GDNF* and *GDNF-AS* transcription start sites (TSSs), reinforced the relationship between both genes and pointed toward the presence of a bi-directional promoter [37,40,41].

We next analyzed the sequence conservation of the dog genomic region encompassing *GDNF/GDNF-AS* (chr4:70,8–71 Mb) and showed that it matches the human genome (chr5:37,8–37,9 Mb) with 84.2% of nucleic acid identity (Fig 3). Interestingly, the sequence surrounding the AMS variant on the human genome overlaps with regulatory regions defined by ENCODE data [36,42] using typical histone marks such as monomethylation of histone H3 at lysine 4 (H3K4me1) and acetylation of histone H3 at lysine 27 (H3K27ac) (Fig 3). These typical histone marks led us to hypothesize [43] that the AMS variant is located in a region which could control *GDNF* and *GDNF-AS* expression.

### Functional analyses

**Fig 4:**
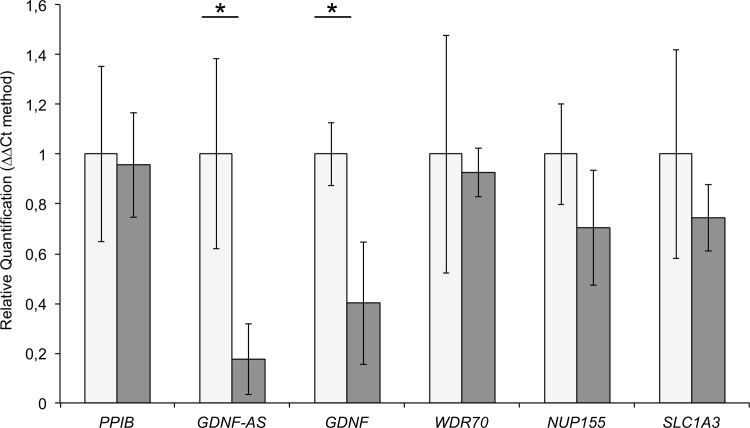
qRT-PCR results in Dorsal Root Ganglia using the ΔΔCt methodology (cases in grey, controls in white). Measures obtained from two DRG by individual were pooled, and we also pooled affected dogs and controls. We used *PPIB* (*Peptidylprolyl Isomerase B*) as control and all genes of the locus (*GDNF-AS*, *GDNF*, *WDR70*, *NUP155*, *SLC1A3*) were tested 3 times with 3 replicates for each (t-test: p-value *<0.0001).

To confirm relationship between *GDNF* and *GDNF-AS*, we performed quantitative real-time PCR analyses on Dorsal Root Ganglia (DRG). Indeed, DRG are interesting, as it was previously recognized as harboring the predominant lesions in these breeds [18,19] and contain sensory neurons where neurotrophic factors are highly expressed [22,23,44–47]. We thus extracted RNAs from DRG of two affected and two unaffected dogs and characterized the expression levels of *GDNF* and *GDNF-AS* together with the neighboring genes *WDR70*, *NUP155*, and *SLC1A3*. QRT-PCR revealed that the *GDNF* mRNA level was significantly decreased (60%) in affected dogs (t-test: p-value *<0.0001). We also showed that the expression levels of the other genes of the locus were not altered (Fig 4). Finally, we also observed a strong decrease of the *GDNF-AS* expression level (80%) in this tissue in agreement with the hypothesis that *GDNF-AS* and *GDNF* share a bi-directional promoter highlighted by correlated expression shown by RNA-seq and genomic analyses (Fig 3, S1 Fig and S5 Table).

The comparative genomic analysis indicates that the region containing the AMS mutation corresponds to an enhancer element (Fig 3). Mutations in regulatory elements are already described in cancer [48,49] or in developmental disorders such as Van der Woude syndrome [50] or isolated pancreatic agenesis [51]. To test the hypothesis of a mutated regulatory element, we constructed reporter systems using a pTAL-Luc plasmid in which the wild-type and mutated sequences were linked to a firefly luciferase reporter gene with a promoter (S2 Fig). While we detected a weak luciferase signal (p-value < 0.05) showing potential enhancer activity with these clones in HeLa cells, no significant difference between cases and controls were observed. However, the distance between the AMS variant and the promoter is ~2kb in these constructs which is a short distance compared to the ~90kb of the genomic region. We thus hypothesized that a long-distance regulation could exist, explaining the qRT-PCR results. The AMS variant region should thus contain regulatory elements sequences.

**Fig 5:**
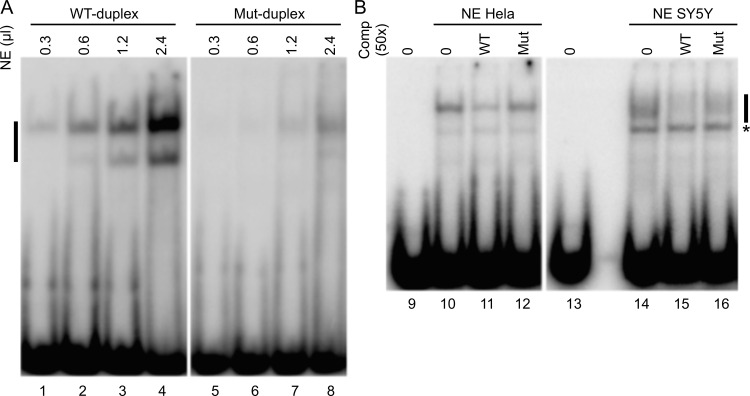
The AMS variant affect binding of nuclear complex. EMSAs were performed with wild type (WT) or mutated (Mut) duplex and nuclear extract (NE). (A) Detection of a mobility shift after incubation of 40 fmoles of radiolabelled WT-duplex (lane 1 to 4) or Mut-duplex (lane 5 to 8) with increasing amounts (0,3–0,6–1,2–2,4 μl) of HeLa NE. (B) 40 fmoles of radiolabelled WT-duplex were incubated without (lane 9 and 13) or with 1μl of HeLa NE (lane 10 to 12) or 5 μl of SY5Y (lane 14 to 16) NE and in the presence of 2 pmol of competitor WT-duplex (lane 11 and 15) or Mut-duplex (lane 12 and 16). Black bar: specific complex associated with the radioactive duplex, asterisk: non specific complex.

To test this hypothesis, we performed Electrophoretic Mobility Shift Assay (EMSA). We first used increasing amounts of nuclear extract from HeLa cells with duplex of wild-type or mutated sequences. We showed a high-affinity specific interaction of a Nuclear Extract (NE) complex with the double-stranded oligonucleotide containing the wild-type sequence but a low-affinity with the sequence containing the AMS variant (Fig 5A). In addition, the competition experiment showed that the complex binds preferentially on the wild-type sequence rather than on the mutated sequence. This result indicates that the affinity of the complexes formed on the two sequences is different, (Fig 5B). The same results were obtained when using human neuroblastoma cell line NE (SY5Y). These data indicate that the mutation prevents the binding of a nuclear complex supporting the hypothesis that the decrease expression of both GDNF and GDNF-AS observed in qRT-PCR experiments (Fig 4) is due to a lack of activity of the mutated enhancer.

## Discussion

This study presents the identification of the mutation involved in a severe form of hereditary sensory autosomal neuropathy (HSAN) shared by four sporting breeds having a common history. The identification of a 1.8 Mb homozygous region on chromosome 4 by GWAS, with only one variant perfectly segregating with the disease, illustrates the advantages of the canine model in genetics. Since this variant is located 90 kb upstream of the neurotrophic factor *GDNF*, we hypothesized a regulatory effect of this variant on *GDNF*. We then showed a strong correlation between the presence of the mutation and the decreased expression levels of both *GDNF* and its divergent long non-coding RNA *GDNF-AS*. Finally, we demonstrated the impact of the mutation on the binding of a complex confirming the hypothesis of a regulatory element. With this work, we provide relevant arguments to explore *GDNF*/*GDNF-AS* partnership in human patients with unexplained HSAN.

We started this project focusing our study on dogs with self-mutilations using a precise clinical questionnaire and we quickly detected that all dogs with self-mutilations also presented insensitivity to pain, not always reported. This important clinical sign led us to improve the genetic analysis. Indeed, we found additional affected dogs without self-mutilations but related to cases with self-mutilations. These dogs showed the “affected” homozygous haplotype, while owners did not detect the insensitivity to pain. This observation reflects the difficulty to diagnose insensitivity to pain in dogs, which contributed to the spreading of this severe disorder in the four related breeds [18,19,21]. This feature led us to consider self-mutilation, probably triggered by small fractures of toes or other injuries [21], as a consequence of the insensitivity to pain.

With a high coverage targeted sequencing of the locus (365x), we identified one variant perfectly associated with the phenotype, and absent in more than 900 unaffected dogs. This variant located upstream of the *GDNF* gene appeared as an excellent candidate. Indeed, *GDNF* (glial cell-derived neurotrophic factor) promotes the axon development and the survival of sensory neurons at different stages of the development in mice and chicken model [22–25]. Moreover, it has been shown *in vitro* that after injury, nearly 100% of the sensory neurons are rescued by the production or injection of *GDNF* [24,46,52,53]. We thus expected that this AMS variant could disturb the expression of *GDNF* leading to the decrease of the number of sensory neurons and then their death, as previously observed in affected dogs [18–20]. This feature could happen during the first months of life, which would correlate well with the early age of onset of this insensitivity to pain.

The genomic analyses of the human and dog orthologous regions revealed the presence of long non-coding RNA transcripts in both species. One of them, *GDNF-AS*, described in human [29], contains the same structure as the canine lincRNAs identified in this study and more likely share a bi-directional promoter with the *GDNF* gene. While the *GDNF* mRNA sequence is well conserved between human, mouse and dog, the canine *GDNF-AS* transcript sequences diverge significantly from the human sequences at the nucleotide level (only 14.3% identity using pairwise alignment with longest isoforms) and no *GDNF-AS* are yet annotated in mouse. However, the whole locus is well conserved at the syntonic and nucleotide levels between human and dog. Regarding shared lncRNAs functions between species in spite of low sequence conservation, several well-described lncRNAs (e.g. *Xist*, *TUNA and Hotair…*) exhibit conserved biological functions even though nucleotide conservation is only limited to small patches and their exon/intron structure is not maintained [54].

An interesting point is the fact that the AMS variant is located in the last exon of *GDNF-AS*, which is the only exon shared by all of the 12 isoforms annotated by our RNA-seq data. Strikingly, another neurotrophic factor, BDNF (Brain Derived Neurotrophic Factor precursor), playing similar functions as GDNF, also presents a similar genomic feature than *GDNF*, with the presence of a lincRNA transcribed in the opposite direction. The parallel between BDNF and GDNF is interesting because they both stimulate the growth and differentiation of new neurons and support the survival of existing neurons in central and peripheral nervous systems [23,52,55]. Moreover, BDNF interacts with the TrkB receptor encoded by the *NTRK2* gene, which has a paralog *NTRK1*, already annotated as mutated in HSAN type IV [5,56,57]. In addition, it has been shown that the inhibition of *BDNF-AS* increases BDNF levels in vivo [38,58]. Modarresi et al. recently showed that the knock-out of *GDNF-AS* increases the expression level of *GDNF* in human HEK293T cells [59]. We could thus hypothesize that the *GDNF* and *GDNF-AS* partnership could fine-tune the expression of *GDNF* and that the AMS variant disturbs this relationship at a specific developmental stage [60].

In embryonic rats, dorsal root ganglia (DRG) neurons were described as potential target of *GDNF* [61,62]. In addition, a recent paper in human and rat confirmed that DRG are the main tissue where genes involved in sensory neuropathies are highly expressed [47]. Thus, the qRT-PCR analyses in canine DRG brought a relevant result: the AMS variant seems to affect a regulatory element. Indeed, while the AMS variant is located in the last exon of *GDNF-AS*, both *GDNF* and *GDNF-AS* transcripts are weakly expressed in affected dogs as compared to unaffected dogs (Fig 4). This important observation supports the implication of GDNF in the disease, and suggests that too low levels of GDNF in the peripheral nervous system prevent the maintenance of the integrity of sensory adult neurons [22–25]. Moreover, this observation led us to consider the hypothesis of a mutated regulatory element that could impair the activation of the promoter shared by *GDNF* and its lincRNA *GDNF-AS*.

While we detected an enhancer activity, we did not find any difference of activity between the wild-type and the mutated sequences in the luciferase reporter assay. This is probably due to the fact that *GDNF* regulation is modulated by a long distance interaction between the region containing the AMS variant and the promoter. As the luciferase reporter assay did not validate this hypothesis, we checked if the AMS variant could inhibit the enhancer activity by preventing the binding of a transcription complex. The EMSA experiment clearly showed that a complex binding the wild-type sequence has less affinity for the mutated sequence. The fact that the mutation of a single nucleotide, in a 20-nucleotides sequence, inhibits the fixation of regulatory elements comforts the causal effect of the variant. It will be interesting to further investigate the nature of the complex as we noticed that the AMS variant introduces a change of one nucleotide in the conserved binding motif recognized by NEUROD1 (S3 Fig). This transcription factor plays an important role during the neurogenesis by binding to regulatory elements of neuronal genes that are developmentally silenced by epigenetic mechanisms [65]. In this context, decreased binding of neurogenic transcription factors to the mutated enhancer in dog could explain the weak expression of *GDNF*/*GDNF-AS* partnership in neuropathies.

While the complete knock-out *GDNF-/-* mouse model is not viable since they die at 1–1.5 days after birth [63,64], the AMS variant is not lethal in dogs. In affected dogs, we showed that *GDNF* and *GDNF-AS* RNAs are still produced but at very low levels. With the functional analyses, we can conclude that the AMS variant affects the regulation of *GDNF* expression, which could explain the non-lethal effect of the mutation in homozygous dogs who only present insensitivity to pain. However, heterozygous dogs are not affected similar to *GDNF+/-* mice. Indeed *GDNF+/-* mice were normal, viable and indistinguishable within controls by visual inspection [64]. Comparison with the mouse model revealed another difference: in dogs, no sign of autonomic disturbances was detected by neurological exams [21] and no metanephric kidney or gastrointestinal tract disorders were observed contrary to what is reported in *GDNF-/-* (knock-out) mice [63,64]. These results confirm that *GDNF* acts in a dose-dependent manner as suggested by *GDNF-/-* mice model [63].

In conclusion, considering the parallel with BDNF function, our RNA expression analyses, and dog/human comparative genomic analyses, we hypothesize that the *GDNF*/*GDNF-AS* partnership represents a very precise regulation mechanism for the mRNA expression of *GDNF*. Indeed, since we showed that the mutation (in the last exon of *GDNF-AS*) dramatically diminishes both the expression of *GDNF*/*GDNF-AS*, by probably disrupting a regulatory element, the AMS variant most probably leads to a decrease of the *GDNF* expression, preventing a correct growth and maintenance of the small fibers from the knees down to the paws. To date, no mutation in the *GDNF* gene or in its regulatory regions has been described in human HSAN patients. This work illustrates the power of the canine model in genetics and shows the potential implication of *GDNF* in such neurological diseases, revealing the importance to explore this gene and its regulatory elements as serious candidates in human HSAN patients.

## Methods

### Ethics statement and sample collection

All dogs were client-owned and no harmful procedures were performed, so there was no animal experimentation according to the European legal definition (Subject 5b and 5f of Article1, Chapter I of the Directive 2010/63/UE of the European Parliament and of the Council). Blood and biopsies were obtained as part of routine clinical procedures for diagnostic purposes, approved by the CNRS ethical board (France), and were sent by veterinarians and French Veterinary Schools. The biological samples were obtained from the ‘Cani-DNA_CRB’, which is part of the CRB-Anim infrastructure, ANR-11-INBS-0003 (http://dog-genetics.genouest.org). The work with dog samples was approved by the CNRS ethical board, France (35-238-13) for UMR6290.

### Samples

Genomic DNA was extracted from 2 mL blood samples collected in EDTA, using the NucleoSpin Blood L kit (Macherey-Nagel) according to the manufacturer’s instructions. Epidemiological and clinical data were collected using a dedicated questionnaire for each affected dog. Dogs over three years old without any symptoms were considered unaffected. Complementary neurological diagnoses (proprioception, motor abilities, spinal reflex exams, electromyography) were done by neurologists, in the Veterinary Schools of Lyon (France) and St-Hyacinthe (Canada).

### Genome-wide association study

Using the Illumina Canine HD 173k (BeadChip), genotyping was performed at the Centre National de Génotypage (CNG; Evry, France) in the frame of European LUPA project in 62 dogs: 49 French spaniels (21 affected and 28 unaffected dogs). This design contained 2 subpopulations: one from France and one from Canada, each including 3 small families (parents + 2 sibling including one affected and one unaffected), all other dogs were unrelated to one another at the grandparent level. To reduce the 3 Mb locus, additional dogs were genotyped including 5 German Short-haired Pointers (1 affected dog, his unaffected brother and father, and 2 other unrelated unaffected dogs), 4 unrelated English Pointers (1 affected and 3 unaffected dogs) and 4 unrelated English Springer Spaniels (1 affected, and 3 unaffected dogs). Using the PLINK software (v1.06–1.07) [66] for SNP filtering (minor allele frequency <0.01; call rate by marker and individual>75%), a dataset of 123,579 SNPs was obtained and analyzed. Genome-wide association study was performed using the software GEMMA v0.94.1 (Genome-wide Efficient Mixed-Model Association) [67,68]. The linear mixed-model method accounts for population stratification and relatedness between dogs, especially for small families.

### Targeted capture by next-generation sequencing and allele-specific PCR

Genomic library sample preparation was performed using the Illumina paired-end library sample preparation kit (Illumina Inc., San Diego, CA, USA). Sample preparation was carried out by Integragen (Evry, France) according to the manufacturer’s instructions (Agilent), using 4μg of genomic DNA. We used two unrelated affected dogs in French Spaniel (FS), one affected German Shorthaired Pointer (GP) and one affected English Springer Spaniel (S1 Table). For controls, we used father of one the two affected FS + one unrelated, one brother of the affected GP and one unrelated ESS. All these dogs were previously genotyped on the Illumina Canine HD 173k. For targeted sequencing, solution-based capture was performed using the Agilent SureSelect Target Enrichment System Kit. Repeated elements were not sequenced due to the limit of this technology, this is why we extended the size of the targeted locus to 3 Mb (chr4: 70.3–73.3 Mb, CanFam3). A custom panel of 75 bases cRNA primers was designed to sequence the 3 Mb locus based on the canine genome (CanFam3 assembly) in the candidate region on chromosome 4. The targeted regions were covered by approximately 25,000 probes designed for 2x coverage (i.e., each base was covered by two different probes). Targets were pulled down via streptavidin magnetic beads, purified, and enriched through 13 cycles of PCR amplification. Samples were paired-end sequenced on an Illumina HiSeq2000. Reads mapping was performed using Bowtie 2 [69] and variants were called using GATK 3.5 [70]. Candidate variants were then genotyped on 100 breeds, each breed corresponding to a pool of four DNA samples of unrelated dogs (S3 Table). We also used small French Spaniel families previously genotyped, to create 12 different pools used as references to detect the presence of the rare allele. Genotyping was performed using an Allele Specific PCR method (AS-PCR) (Integragen) developed from the methodology described by Nazarenko and Myakishev [71,72]. SNP genotyping was performed on the Biomark (Fluidigm) with a microfluidic multiplex 96.96 dynamic array chip. We excluded all variants where rare alleles were found in minimum three different breeds (minimum one dog by breed).

### Identification of the candidate variant in dogs

All primers used were designed using Primer3plus (www.bioinformatics.nl/primer3plus/) [73]‎. We re-sequenced the *GDNF* gene and the variant on 16 affected and 16 unaffected French Spaniel using the Type-it Microsatellite PCR kit (Qiagen) on C1000 and S1000 thermocyclers (Bio-Rad). PCR products were cleaned using the Illustra Exostar-1 Step reagent (Dutscher) and sequenced by Sanger method using the BigDye Terminator v3.1 cycle sequencing kit (Thermo Fisher Scientific). Electrophoresis of the products was realized on a 370 ABI sequencer. Sequences were analyzed using the PhredPhrap and Consed software’s [74–76]. The presence or absence of the variant was validated on 250 French Spaniel, German-Short-Haired Pointer, English Pointer and English Springer Spaniel (24 affected and 226 unaffected), and 900 dogs (including the 400 dogs used for AS-PCR) from 130 unaffected breeds among the 10 FCI (Fédération Cynologique Internationale) groups.

### RNA sequencing analyses and *GDNF-AS* annotation

To detect the *GDNF-AS* long non-coding RNA (lincRNA), we analyzed 33 RNA sequencing data produced from 25 different tissues of 7 breeds (S4 Table) as a part of the LUPA consortium and the Broad Institute annotation efforts [31]. Each RNA sequencing data includes between 60 and 150 millions paired-end and strand-specific reads respectively, that were analyzed by Bowtie/Tophat2 and CUFFLINKS/CUFFMERGE v2.2.1 [69,77] revealing 245,276 transcripts belonging to 81,363 genes. The protein-coding capabilities of all novel transcripts (*i*.*e*. not annotated in Ensembl) was measured using three complementary programs CPC, CPAT, PLEK [33–35] (S1 File). We quantified RNA expression at the gene-level using the HTSEQ-count program version 0.6.1 [78] where each gene is considered as the union of its exons. This gene-count based measure was then transformed in RPKM using the edgeR software version 3.14.0 [79] in order to normalize gene expression by both its effective length and the total number of mapped reads (thus the sequencing depth) in each of the 33 samples [80]. Therefore, each gene is assigned a vector of 33 points corresponding to the normalized expression of the gene in the 33 RNA-seq samples. Using the cor.test function of the R software (https://www.r-project.org), we computed all pairwise Pearson correlations for the 5 representative genes in the AMS locus i.e. *GDNF-AS*, *GDNF*, *WDR70*, *NUP155*, and *C5orf42* (S1 Fig) based on the 33 points.

### Candidate gene expression analysis

RNA was extracted from tissues, using the NucleoSpin RNA kit (Macherey-Nagel) according to the manufacturer’s instructions. We used two affected French Spaniel (brother and sister) and two unrelated dogs for controls, all already genotyped on the Illumina Canine HD 173k. Reverse transcription was performed on 1 μg of total RNA using the High-capacity cDNA Reverse Transcription kit (Thermo Fisher Scientific), according to the manufacturer's instructions. The total Dorsal Root Ganglia cDNA of the canine *GDNF* and *GDNF-AS* were amplified and sequenced using two primer pairs (S6 Table). qRT-PCR was performed on 1:80 diluted cDNA samples after pre-amplification with the TaqMan PCR master mix (Thermo Fisher Scientific) on the 7900HT Fast Real-Time PCR System (Applied Biosystems) using standard procedures. We used pre-designed primers for *GDNF* (Thermo Fisher Scientific Reference: Cf03986046_g1), *WRD70* (TFS Ref: Cf02651565_m1), *NUP155* (TFS Ref: Cf02644933_m1) and *SLC1A3* (TFS Ref: Cf02702629_m1) and we also specifically designed other probes for *GDNF-AS* (S6 Table). Canine *PPIB* (*Peptidylprolyl Isomerase B*) was used as the reference gene (Thermo Fisher Scientific Reference: Cf02629556_m1). Each sample was measured in triplicate and each qRT-PCR was carried out three times by different experimenters. Relative amounts of the transcript were determined using the ΔΔCt method [81]. Expression analyses were carried out on two tissues (DRG L6, DRG L7) and their results were pooled. We also pooled results from both affected dogs and both controls to increase the number of points. Using the t.test function of the R software (https://www.r-project.org), we determined the significance of the variation of expression level for all genes.

### Cloning and luciferase assays

Clones were created to be centered on the AMS variant and correspond to a fragment of 2359 bp containing two SINE, two LINE, one LTR, and eight SNVs. PCR was performed from the genomic DNA of two dogs: one affected dog carrying the mutation and rare alleles for 8 SNVs, and one unaffected dog with the same haplotype as the dog genome reference. The PCR fragments were cloned into the pTAL Luciferase plasmid (Clontech Laboratories, Inc.) by DNA ligation or by homologous recombination using the Gibson assembly MasterMix (New England, Biolabs). All primer sequences used to prepare these constructs are given in S6 Table. All constructs were verified by DNA sequencing. Hela cells were grown in high-glucose Dulbecco’s Modified Eagle’s Medium (DMEM) containing 10% fetal calf serum (GIBCO) according to the manufacturer’s instructions. Cells were transfected in triplicates with 250 ng of reporter plasmid and 50 ng of Renilla control vector (pRL-Null from Promega) using JetPEI (Polyplus Transfection) in 24-well plates. After 24 h, Firefly and Renilla luciferase activities were determined using the Dual-Luciferase Reporter Assay System (Promega) on a Veritas Microplate Luminometer (Turner Biosystems). Enhancer assays of selected regulatory regions were run as previously described [82]. Firefly luciferase activities of individual transfections were normalized against Renilla luciferase activities. As positive control (control +), we used a home-made construction with enhancer activity previously described by Sérandour *et al* [82].

### Cell culture and nuclear extracts

HeLa nuclear extract were purchased from the Computer Cell Culture Center S.A. (Belgium) and prepared according to Dignam et al [83]. SY5Y cells were grown in 50% Dulbecco's Modified Eagle Medium (DMEM) and 50% Nutrient Mixture F-12 with 10% of fetal calf serum and 1% of antibiotics and maintained at 37°C and 5% CO2. Cells were harvested using 1 ml of Lysis buffer containing protease inhibitors, 10 mM Tris-HCl (pH 7.5), 0,5% NP40, 2 mM MgCl2, 3 mM CaCl2 and 10% Glycerol and gently spun down. The supernatant was removed, and the cells were resuspended in the lysis buffer and gently spun down. The supernatant was removed and the cells were resuspended in 500ul of Extraction buffer containing protease inhibitors, 20 mM Hepes, 0,4 M KCL, 1,5 mM MgCl2, 2 mM DTT, 0,5 mM PMSF and 20% Glycerol. The cells were left on ice for 30 min to swell and were then vortexed vigorously for 10 s once during the time on ice. The cells were centrifuged at 12,000 rpm, 15 min at 4°C. The supernatant was kept and the protein concentration was subsequently estimated by the BCA assay.

### Electrophoretic Mobility Shift Assays

Probe labeling and duplex formation: 10 pmoles of primers wild type sequence (WT: 5’- TGTTGTCTTTGCTGCTGTCATGATGG-3’) and mutated sequence (Mut: 5’- TGTTGTCTTTGCTACTGTCATGATGG-3’) were 5'-end labeled using T4-PNK (Promega M4101) and 20μCi of ^32^P-g ATP. Labeled oligonucleotides were purified on a Sephadex G-25 column. Unlabeled complementary oligonucleotides were then annealed to form a WT-duplex or Mut-duplex in hybridization buffer (10mM Tris pH7.4; 6mM MgCl_2_; 50mM NaCl; 6mM B-Mercapto-Ethanol) by incubation for 1 min at 95°C and gradually cool down to room temperature. Complete annealing of the radioactive probe was checked by electrophoresis on 20% native acrylamide gel. For complex formation, 40fmoles (15000cpm) of duplex was incubated with various amount of nuclear extracts in a total volume of 10μl containing 25 mM HEPES (pH 7.5), 150 mM KCl, 5 mM dithiothreitol (DTT), 10% glycerol, 1 μg of poly (dI-dC). Incubation was performed for 30 min on ice prior to loading. For competition experiments, the mixture was preincubated for 30 min at 4°C with 2 pmoles of unradiolabeled competitor duplex, before addition of the radiolabeled duplex. Complexes were then fractionated onto a 5% polyacrylamide (38:2) gel with 2.5% (v/v) glycerol, 0.5 mM EDTA and 22.25 mM Tris-borate (pH 8.3) by electrophoresis at 4°C in 0.5 mM EDTA, 0.25%glycerol and 22.25 mM Tris-borate (pH 8.3). Free and bound duplexes were visualized with a phosphoimager using the ImageJ software.

## Supporting Information

S1 File Fasta files of the canine *GDNF-AS* sequences of the 12 isoforms. (TXT)

S1 Fig Heatmap of the Pearson correlations between *GDNF-AS*, *GDNF*, *WDR70*, *NUP155* and *C5orf42* expressions. (PDF)

S2 Fig Functional analyses of the candidate regulatory element. (PDF)

S3 Fig Canine sequence with a conserved NEUROD1 motif binding site (PDF)

S1 Table Description of the targeted sequencing using Agilent technologies. (XLSX)

S2 Table List of the 156 variants detected by targeting sequencing. (XLSX)

S3 Table Lists of the different breeds used to check candidate variants. (XLSX)

S4 Table Lists of the different tissues used for the RNA sequencing. (XLSX)

S5 Table Expression values (RPKM) for the Acral Mutilation Syndrome locus and candidate receptors. (XLSX)

S6 Table Primers used for Sanger sequencing, qRT-PCR and cloning. (XLSX)
