# Supplementary figures and images for: A Point Mutation in a lincRNA Upstream of GDNF Is Associated to a Canine Insensitivity to Pain: A Spontaneous Model for Human Sensory Neuropathies
Source: PLoS Genet. 2016 Dec 29;12(12):e1006482. doi: 10.1371/journal.pgen.1006482 (PMC5198995; doi:10.1371/journal.pgen.1006482)

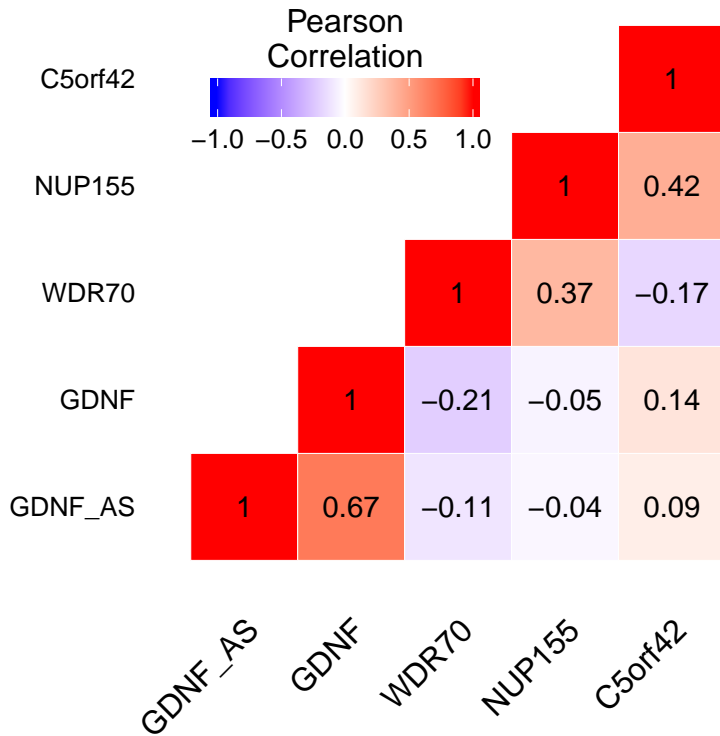

Supplement: S1 Fig — (PDF) [file pgen.1006482.s002.pdf]
