# Supplementary material for: A Point Mutation in a lincRNA Upstream of GDNF Is Associated to a Canine Insensitivity to Pain: A Spontaneous Model for Human Sensory Neuropathies
Source: PLoS Genet. 2016 Dec 29;12(12):e1006482. doi: 10.1371/journal.pgen.1006482 (PMC5198995; doi:10.1371/journal.pgen.1006482)
Supplement: S2 Fig — (PDF) [file pgen.1006482.s003.pdf]

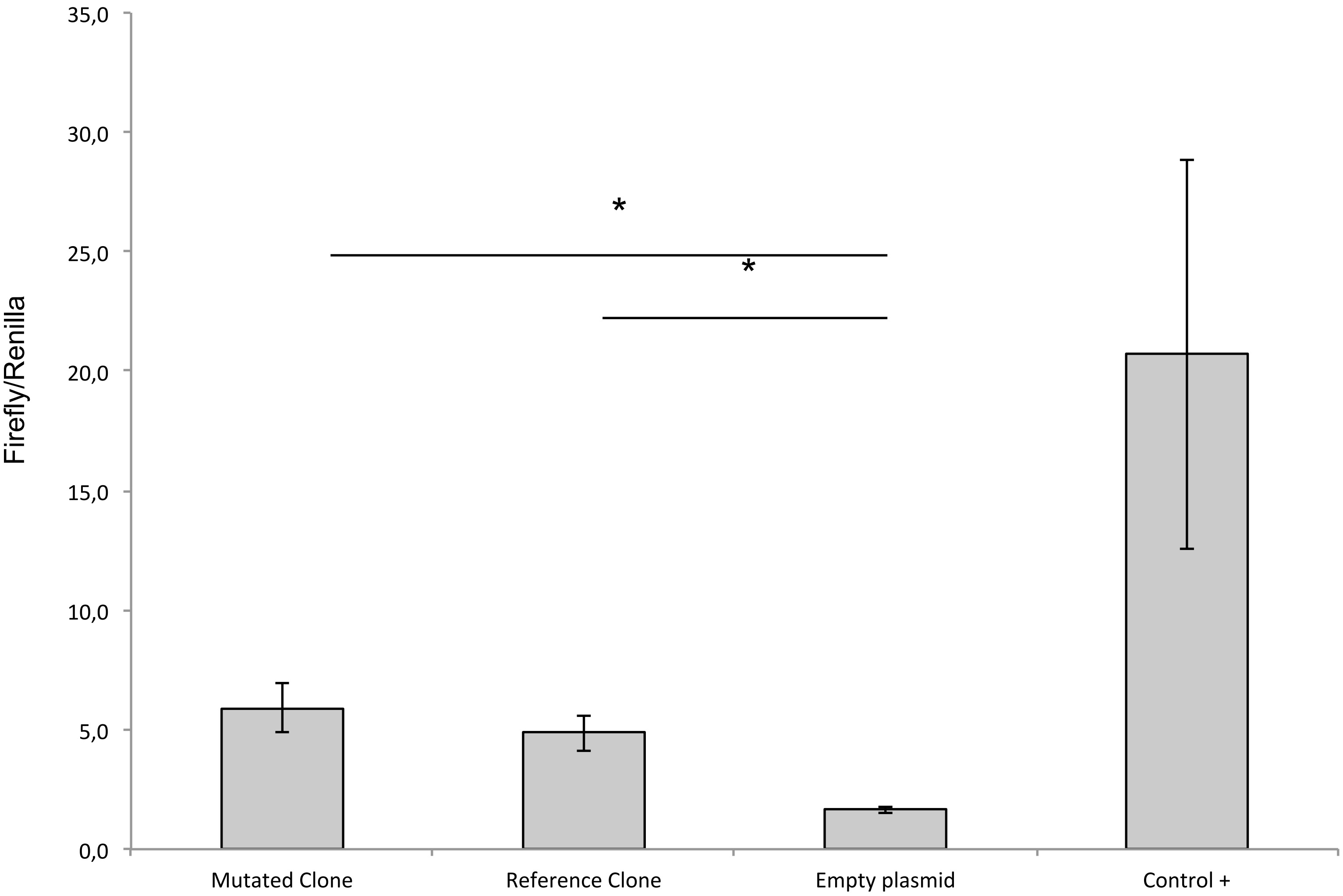


**S2 Fig. Functional analyses of the candidate regulatory element.**

We constructed reporter systems using a pTAL-Luc plasmid in which the wild-type and mutated sequences were linked to a firefly luciferase reporter gene with a promoter in HeLa cells. Y axis is the Firefly Luciferase/Renilla ratio. Control + corresponds to a clone with an enhancer activity used in a previous study [82]. No difference between the mutated clone and the reference clone are observed but we noticed significant differences with the control (empty plasmid) suggesting an enhancer activity (Student-test p-value: *<0.05).
