# Supplementary material for: A Point Mutation in a lincRNA Upstream of GDNF Is Associated to a Canine Insensitivity to Pain: A Spontaneous Model for Human Sensory Neuropathies
Source: PLoS Genet. 2016 Dec 29;12(12):e1006482. doi: 10.1371/journal.pgen.1006482 (PMC5198995; doi:10.1371/journal.pgen.1006482)
Supplement: S3 Fig — (PDF) [file pgen.1006482.s004.pdf]

S3 Fig. Canine sequence with a conserved NEUROD1 motif binding site

5′--GTCTTTGCTGCTGTCATGATGAT--3′

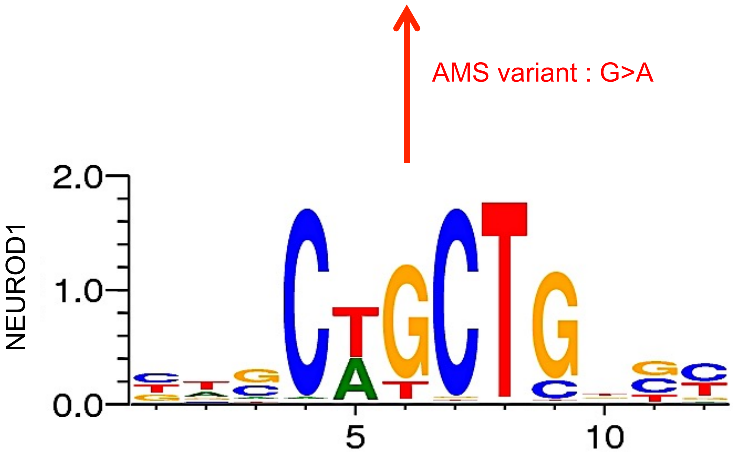

NEUROD1 motif defined by MotifMAp (Daily et al, 2011) matches perfectly with the sequence. The substitution of a guanine by an adenine in the third conserved nucleotide of this motif could disrupt the binding with NEUROD1 in affected dogs.

Daily K, Patel VR, Rigor P, Xie X, Baldi P. MotifMap: integrative genome-wide maps of regulatory motif sites for model species. BMC Bioinformatics. BioMed Central Ltd; 2011;12: 495. doi:10.1186/1471-2105-12-495
